# Targeting ligand-gated ion channels in neurology and psychiatry: is pharmacological promiscuity an obstacle or an opportunity?

**DOI:** 10.1186/1471-2210-10-3

**Published:** 2010-03-02

**Authors:** Matt T Bianchi, Emmanuel J Botzolakis

**Affiliations:** 1 Neurology Department, Sleep Division, Massachusetts General Hospital, Boston, MA, USA; 2 Medical Scientist Training Program, Vanderbilt University School of Medicine, Nashville, TN, USA

## Abstract

**Background:**

The traditional emphasis on developing high specificity pharmaceuticals ("magic bullets") for the treatment of Neurological and Psychiatric disorders is being challenged by emerging pathophysiology concepts that view disease states as abnormal interactions within complex networks of molecular and cellular components. So-called network pharmacology focuses on modifying the behavior of entire systems rather than individual components, a therapeutic strategy that would ideally employ single pharmacological agents capable of interacting with multiple targets ("magic shotguns"). For this approach to be successful, however, a framework for understanding pharmacological "promiscuity" - the ability of individual agents to modulate multiple molecular targets - is needed.

**Presentation of the Hypothesis:**

Pharmacological promiscuity is more often the rule than the exception for drugs that target the central nervous system (CNS). We hypothesize that promiscuity is an important contributor to clinical efficacy. Modulation patterns of existing therapeutic agents may provide critical templates for future drug discovery in Neurology and Psychiatry.

**Testing the Hypothesis:**

To demonstrate the extent of pharmacological promiscuity and develop a framework for guiding drug screening, we reviewed the ability of 170 therapeutic agents and endogenous molecules to directly modulate neurotransmitter receptors, a class of historically attractive therapeutic targets in Neurology and Psychiatry. The results are summarized in the form of 1) receptor-centric maps that illustrate the degree of promiscuity for GABA-, glycine-, serotonin-, and acetylcholine-gated ion channels, and 2) drug-centric maps that illustrated how characterization of promiscuity can guide drug development.

**Implications of the Hypothesis:**

Developing promiscuity maps of approved neuro-pharmaceuticals will provide therapeutic class-based templates against which candidate compounds can be screened. Importantly, compounds previously rejected in traditional screens due to poor specificity could be reconsidered in this framework. Further testing will require high throughput assays to systematically characterize interactions between available CNS-active drugs and surface receptors, both ionotropic and metabotropic.

## Background

A common assumption underlying drug discovery is that therapeutic agents with higher specificity for their molecular targets confer better efficacy and fewer side effects. Indeed, drug discovery efforts traditionally focus on developing "magic bullets" - agents that provide the proverbial surgical strike against critical players in a disease process while minimizing collateral damage. However, there is growing interest in the possibility that drug promiscuity (defined as clinically meaningful interaction between a drug and multiple molecular targets) may actually represent a therapeutic benefit rather than a liability. If true, then screening for "magic shotguns" - therapeutic agents that are rationally promiscuous - could be a more effective drug discovery strategy [1-6]. This concept is supported by both theoretical and empirical studies, and is congruent with our current understanding of biology in general: that is, genes, proteins, and signaling molecules are multi-functional and comprise a complex network of interactions[3,4,7-9]. Insight into the effects of therapeutic agents upon these networks has been fueled by the recent explosion in genomic and proteomic investigations, which have elucidated the complex molecular interactions in disease states[9]. Similarly, protein-protein interaction networks have yielded elaborate datasets from organisms spanning yeast, nematodes, and humans, revealing novel sets of potential therapeutic targets for disease processes [10-12].

## Presentation of the Hypothesis

For disorders of the central nervous system (CNS), where highly complex interactions underlie normal function, drug promiscuity may be particularly relevant. Drug promiscuity is already well-recognized among certain classes of CNS-active modulators such as general anesthetics [13,14], anticonvulsants [15], and antipsychotics - and this property may extend to other therapeutic classes such as anti-dementia drugs [16] and even purportedly high-specificity agents such as selective serotonin re-uptake inhibitors (SSRIs) [17,18]. However, it remains uncertain which subset of promiscuous interactions is important for clinical efficacy. Potential contributors to this uncertainty include the fruitful history of linking off-target interactions with side effects, as well as the emphasis on high specificity compounds in drug development. Despite the clear importance of off-target interactions with side effects, many drugs acting in the CNS (including some purported to have high specificity) have been shown to interact with multiple targets at therapeutically relevant concentrations. One approach to potentially harness promiscuity as a tool for drug discovery is to ascertain which targets are common among different drugs in a therapeutic class, thereby enriching for the subset of interactions most likely to be therapeutically relevant. We hypothesize that drug discovery strategies developed to screen for such "rational promiscuity" may reveal novel compounds with therapeutic efficacy in diseases of the CNS.

## Testing the Hypothesis

To demonstrate that CNS drugs are generally promiscuous agents and illustrate how mapping promiscuity can guide drug discovery, we collected published examples of acute, direct functional modulation of ligand-gated ion channels (LGICs) by a total of 170 pharmaceutical and endogenous molecules, as demonstrated by *in vitro *electrophysiology. Although indirect (signal transduction) and subacute/chronic (plasticity, gene expression) effects also likely contribute to the clinical efficacy of many CNS-active drugs, we focused only on direct functional interactions demonstrated with physiological measurements (ion flux and binding data were considered insufficient). Although the actions of these 170 modulators on the selected LGICs may or may not be functionally relevant *in vivo*, the degree of promiscuity illustrates the capacity of this class of protein targets to interact with diverse compounds. The potential drug discovery impact of recognizing clinically relevant promiscuity includes the idea that compound library molecules previously rejected on account of poor specificity by *in vitro *screening could be reconsidered in the context of "rational promiscuity".

### The ligand-gated ion channel family: evidence for promiscuous modulation

**Figure 1:**
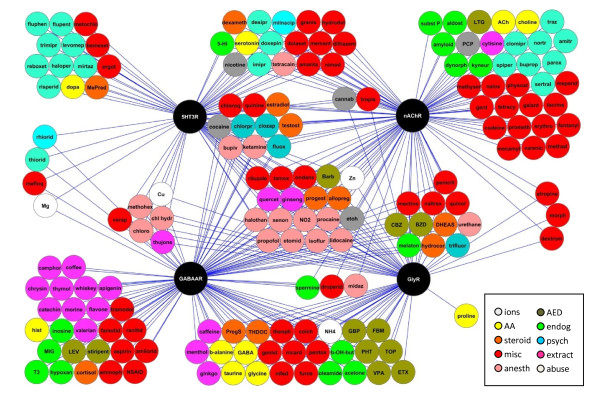
**Promiscuous modulation of ligand-gated ion channels**. The four members of the cys-loop family of ligand gated ion channels are shown as black nodes. Endogenous and exogenous modulators exhibiting electrophysiologically confirmed modulation of one or more of these channel classes are indicated by a blue line connecting the modulator to the receptor(s). Modulators in the four corners are those showing documented modulation of only one channel class, while those centrally located modulate all four classes. *Endog*, endogenous; *AA*, amion acids; *misc*, miscellaneous; *anesth*, anesthetics; *AED*, anti-epileptic drug; *psych*, psychiatric. This figure was generated using Cytoscape software.

Figure 1 illustrates the promiscuous modulation of four LGICs (GABA_A_, acetylcholine, glycine, and 5HT-3 receptors) by 170 compounds identified in a systematic manual search of the PubMed database between 1970 and 2008, as demonstrated by *in vitro *electrophysiology studies. These compounds span categories of psychiatric medications (Additional File 1: Supplementary Table S1), anesthetics (Additional File 1: Supplementary Table S2), anticonvulsants (Additional File 1: Supplementary Table S3), natural extracts (Additional File 1: Supplementary Table S4), amino acids and ions (Additional File 1: Supplementary Table S5), steroids (Additional File 1: Supplementary Table S6), endogenous substances (Additional File 1: Supplementary Table S7), drugs of abuse (Additional File 1: Supplementary Table S8), miscellaneous medications (Additional File 1: Supplementary Table S9). The EC_50_, IC_50_, and modulation percentage are referenced in these tables.

Of the 170 compounds, 60% were active at GABA_A _receptors, 56% at nicotinic acetylcholine receptors, 40% at glycine receptors, and 40% at 5HT-3 receptors. 42% of the compounds interacted with only one LGIC (located in the four corners of Figure 1), 30% interacted with two LGICs, 16.5% interacted with three LGICs, and 11% interacted with all four LGICs (located in the center of Figure 1). Additional connections may exist, as not every molecule was tested systematically across these four LGICs. Modulators spanned several categories, including endogenous species (e.g., amino acids, ions, steroids) and exogenous molecules (e.g., psychotropic, anticonvulsant, anesthetic, and other FDA-approved medications). While some of these interactions are thought to be responsible for clinical efficacy (such as benzodiazepines potentiating GABA_A _receptors) or side effects (such as antibiotics antagonizing GABA_A _receptors), the physiological relevance (if any) of many of these interactions is unknown. However, the extent of promiscuity emphasizes the need for systematic characterization if the hypothesis is to be investigated for drug discovery purposes. Since the evidence for modulation was derived from a wide variety of sources not explicitly designed to test for promiscuity, meaningful comparison of the affinity and efficacy of modulation was not possible (values are nevertheless shown in Supplemental Tables, Additional file 1).

It is worth noting that each of these LGICs is actually comprised of multiple subtypes, each having distinct functional and pharmacological properties. Similar to a recent pharmacological interaction network based on databases of FDA-approved drugs and their targets [19], we have not presented receptor sub-types, which can have profound influence on modulation. For example, GABA_A _receptors are assembled as heteropentamers from a neuronal repertoire of 19 subunit genes, and subunit composition is known to influence modulation[20]. The level of complexity further increases when the promiscuous nature of the neurotransmitters themselves is considered. For example, in addition to binding to GABA_A _receptors, the neurotransmitter GABA binds to two additional receptor classes (metabotropic GABA_B _and ionotropic GABA_C _receptors), as well as to a subset of glycine receptors *in vivo *[21,22]. In fact, the ability of classical neurotransmitters to interact directly with non-canonical targets is well-recognized: glycine is a co-agonist for NMDA-type glutamate receptors, serotonin can activate nicotinic acetylcholine receptors, and dopamine is an agonist at serotonin receptors. Moreover, experimental measurements of modulation are themselves influenced by receptor properties such as agonist affinity, efficacy, and desensitization [23,24]. Modulation mechanisms can be influenced by the modulator concentration: neurosteroids and barbiturates allosterically modulate GABA_A _receptors at low concentrations, act as direct agonists as higher concentrations, and open channel blockers at even higher concentrations concentrations [25-27]. Direct receptor-receptor interactions, independent of second messenger systems, also contributes to complexity [28-31]. Local drug concentration profiles may also exhibit complex regulation within and outside synapses, or via lipid rafts that alter their effective concentration [32].

### Drug-centric promiscuity maps

**Figure 2:**
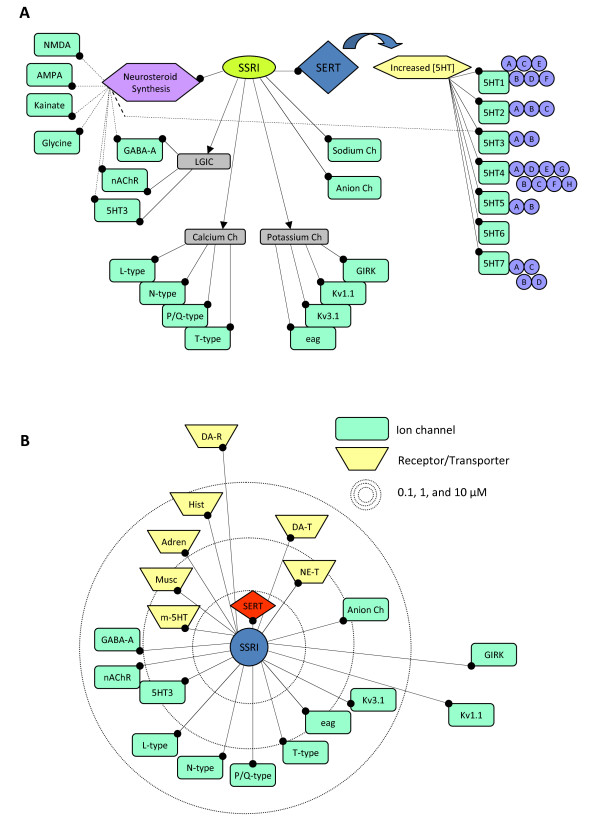
**Promiscuity maps**. A. SSRIs interact with multiple classes of ion channels, and increases neurosteroid synthesis (by directly modulating a rate limiting enzyme activity), in addition to its activity on the serotonin reuptake transporter (SERT). Neurosteroids are themselves promiscuous modulators of ion channels, and some of these interactions are shown (dotted lines from purple box). The impact of increasing serotonin is manifest at potentially any of seven categories of serotonin receptor (each with several subtypes), spanning ionotropic, metabotropic, varying localization, and different second messenger cascades. B. Promiscuity map of direct interactions of SSRIs with various metabotropic receptors and transporters (polygons), as well as ion channels (rectangles) including K channels (blue), Ca channels (green), LGICs (yellow). Distance from the central SSRI node approximates the log-scaled affinity of SSRI for the various targets. Concentric dotted circles reflect 3 orders of concentration magnitude (0.1, 1, and 10 μM). Therapeutic SSRI concentration in vivo is between 1-10 μM, that is, between the middle and outer circle. This figure was generated using CellDesigner software.

Drug promiscuity can also be evaluated from the reverse perspective, focusing instead on the ability of a single therapeutic agent to interact with multiple protein targets. The most comprehensive approach, illustrated in Figure 2A using the example of SSRIs, is to include both direct and indirect targets. Although classically thought to be highly selective agents, three points are immediately apparent from the SSRI map. First, promiscuity exists at the level of direct interactions of SSRIs with CNS targets [17]. Second, one of the direct targets, the rate-limiting step in neurosteroid biosynthesis, leads to the production of additional promiscuous modulators, each of which affects various downstream targets. Finally, the SSRI-induced increase in serotonin concentration potentially impacts numerous serotonin receptor subtypes, spanning multiple signaling processes (pre- and post-synaptic, excitatory and inhibitory, ionotropic and metabotropic, and different second messenger systems).

For drug screening purposes, however, mapping only direct interactions may be more explicitly useful. An example is shown in Figure 2B, where the direct modulation of multiple targets by SSRIs is illustrated. The EC_50 _or IC_50 _of SSRIs for each target is approximated by the radial distance from the center of the map (based on electrophysiology data for the ion channel targets and on binding data for the metabotropic target receptors). The radius of the outer two concentric circles spans the range of estimated therapeutic CNS concentration (1-10 μM) [17]. With this approach, the extent of drug promiscuity can easily be visualized in reference to therapeutically relevant concentrations. Of note, while the affinity of SSRIs for the primary target, the serotonin transporter, is ~50 nM, the therapeutic CNS concentration range in humans is estimated to be more than 10-fold higher, raising the possibility of clinically important interactions with numerous other targets. It should also be emphasized that affinity does not entirely capture the potential for significant interaction. For example, although the EC_50 _for fluoxetine enhancement of GABA_A _receptors was 128 μM, the enhancement was large (350%), suggesting modulation could occur at concentrations as low as 10 μM[33]. Furthermore, the major metabolite norfluoxetine was over 100× more potent in the same study (EC_50 _0.7 μM).

### Using promiscuity mapping to guide drug discovery

**Figure 3:**
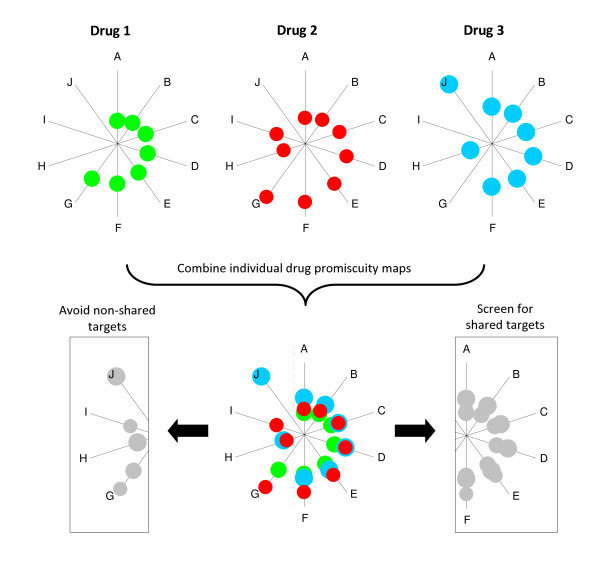
**A rational promiscuity approach to drug screening**. Coverage maps are generated for individual drugs, here plotted as relative affinity for targets A-J indicated by radial distance from the origin (in arbitrary units). Combining the maps clearly illustrates that targets A-F are shared by all 3 drugs (dotted line). Subsequent screening algorithms would enrich for targets A-F, while avoiding non-shared targets G-J.

Using promiscuity maps to guide drug discovery could proceed as follows. First, a drug-centric promiscuity map akin to that shown in Figure 2B would be generated for each drug in a therapeutic class, possibly grouping drugs with similar clinical efficacy (Figure 3). Then, targets shared by all members of the class would be identified, thus representing the subset of interactions presumably linked to the desired clinical effect. Non-shared targets would also be identified, as these are presumably less therapeutically relevant and/or responsible for drug-specific side effects. Screening (or design) of "rationally promiscuous" drugs would then aim to reproduce the shared interactions across similarly efficacious compounds, while avoiding non-shared targets (Figure 3). Granted, this strategy may be somewhat oversimplified since side effects could be shared and thus selected for in such a promiscuity analysis. Additionally, it is conceivable that targets not shared by all members of a class could be relevant for individual patient differences in efficacy seen clinically. However, these are both potentially testable hypotheses through systematic analysis of promiscuity, followed by rational design of drugs exhibiting distinct coverage maps. The currently sparse experimental coverage of possible interactions precludes even preliminary estimates of shared targets. Advances in high throughput electrophysiology will facilitate implementation of this screening strategy.

## Implications of the Hypothesis

The *in vivo *cellular and molecular milieu involves complex interactions between cellular and molecular components. However, because these components are typically studied experimentally in isolation, it remains largely uncertain how such complexity influences signaling during normal brain function or disease states. Moreover, few studies have endeavored to explore the effect of simultaneous endogenous and pharmacological modulators acting on a receptor target, mainly because of the cumbersome combinatorial nature of studying even a subset of the possible interactions. Given the evidence for promiscuity of endogenous as well as therapeutic modulators of neurotransmitter receptors highlighted here, we propose that such information may be biologically and clinically relevant. Indeed, by aiming various high throughput functional assays toward the systematic elucidation of promiscuity maps, we anticipate that new vistas of drug discovery will be revealed. One important implication of this strategy is that one can re-investigate existing compounds that have been discarded in early preclinical studies solely on the basis of failing high specificity criteria.

### The magic shotgun approach to complex disease processes

Three lines of reasoning argue against the assumption that higher specificity drugs will necessarily confer increased therapeutic efficacy. First, endogenous receptors and signaling molecules are themselves multi-functional, exhibiting both ligand-centric and receptor-centric promiscuity. Thus, even theoretically high specificity agents could have unpredictable biological impact, if the intended target were involved in many functions. Second, target promiscuity may be more common than recognized for many CNS-active therapeutics[14,15,17,18], raising the possibility that promiscuity in fact contributes to the clinical efficacy. Finally, there is growing evidence that complex networks are more optimally modulated by multi-target approaches [8], suggesting a paradigm shift from the dominant "magic bullet" strategy to what has become known as the "magic shotgun" approach to therapy[3,4,7,34]. Indeed, to treat most CNS disorders, drugs must limit pathological neuronal activity without disrupting the rich underlying functionality of the brain. The existing evidence for promiscuity raises the possibility that modulating multiple nodes of a network of interacting components may be more appropriate for the complex pathophysiology of CNS diseases. An intriguing speculation is that highly specific drugs could invoke greater compensatory homeostatic processes, which may relate to tolerance and/or to side effects. Is it possible that a multi-pronged modulation strategy, with each prong perhaps providing a relatively small effect (that evades large homeostatic cellular responses), would not only be more efficacious, but potentially also reduce side effects? Recent advances in imaging large networks of neuronal firing may shed light on the network-level impact of drugs thought to interact with multiple targets in clinically relevant situations such as depression models [35].

### Promiscuity and clinical efficacy of CNS-active drugs

Rational approaches to promiscuity may inform potential drug discovery strategies that consider the patterns of target modulation in relation to pharmaceutical efficacy as well as side effects. Endogenous signal transduction pathways, such as G-protein coupled receptors and receptor kinases, likely also demonstrate modulator promiscuity, and should be considered in addition to ion channel targets. Several models have been proposed to address the potential molecular basis of target-receptor promiscuity based on atomic-level interactions [36], as well as the propensity for promiscuous versus specific protein-protein interactions in "crowded" cellular environments [37]. Other analysis, arguing in favor of the importance of specificity, suggested that promiscuity was more commonly evident in drugs that did not reach the clinic[38], perhaps due to side effects. Finally, a recent report used ligand-receptor interaction models to explore previously unknown "off-target" interactions experimentally [5].

The fundamental challenge of modern CNS pharmacology is to determine which aspects of drug promiscuity contribute to clinical efficacy, and which aspects are responsible for unwanted side effects[39]. To gain novel insight into normal and pathological physiology as well as potential therapeutic interventions, a multidisciplinary effort is required that combines high throughput techniques and informatics approaches. Rational exploration of the promiscuity parameter space represents one strategy for improving drug discovery by using existing drug classes as a template for future rational design.

## Competing interests

The authors declare that they have no competing interests.

## Authors' contributions

MTB and EJB contributed equally to conceiving the study and its design, interpretation, figure development, and drafting of the manuscript. MTB carried out the primary literature analysis. Both authors read and approved the final manuscript.

## Supplementary Material

Additional file 1 **Tables of modulator-target interactions**. EC_50_, IC_50_, percent modulation, and references for each modulator-target interaction. Click here for file
