# Supplementary material for: Targeting ligand-gated ion channels in neurology and psychiatry: is pharmacological promiscuity an obstacle or an opportunity?
Source: BMC Pharmacol. 2010 Mar 2;10:3. doi: 10.1186/1471-2210-10-3 (PMC2838756; doi:10.1186/1471-2210-10-3)
Supplement: Additional file 1 — Tables of modulator-target interactions. EC50, IC50, percent modulation, and references for each modulator-target interaction. [file 1471-2210-10-3-S1.DOC]

**Additional File: Tables of modulator-target interactions**

Each table shows a class of modulators, which are listed alphabetically. The numbers in each cell are the EC_50_ or IC_50_ values for the given modulator, with the % change shown in adjacent parentheses. Pink shading = inhibition, Green = enhancement, Blue = mixed, Yellow = agonist activity. For enhancement, % value reflects the current increase above control levels, ie, 100% indicates a doubling of current amplitude.

* indicates only a single concentration was used (or insufficient range to calculate EC_50_ or IC_50_).

Abbreviations: *Desens*, modulator increased desensitization of currents; *n/a* = not available; *NO2*, nitrous oxide; *DHEAS*, dehydroepiandrosterone; *THDOC*, tetrahydrodeoxycorticosterone; *PCP*, phencyclidine

Table S1. Psychotropics (n=26)

| **Modulator** | **GABA_A_** | **GlyR** | **nAChR** | **5-HT3** |
| --- | --- | --- | --- | --- |
| Amitriptyline |  |  | 0.5-15 (50-75%)[1-3] |  |
| Bupropion |  |  | 1-40 (50-90%)[4] |  |
| Chlorpromazine | 230-2900 (30-90%)[5-6] |  | 1 (90%)[7] | 0.2 (90%)[8] |
| Clomipramine |  |  | 1.5 (90%)[9] |  |
| Clozapine | 8.2 (50%)[10] |  | 3.2-10 (80-90%)[11-12] | 0.01-1 (90%)[13-14] |
| Desipramine |  |  | 0.2-3 (75%)[15] | 1.1 (90%)[16] |
| Doxepin |  |  | 0.5-15 (75%)[1] | 7 (90%)[17] |
| Fluoxetine | 0.7-128 (200-350%)[18] |  | 0.3-5.3 (90%)[19-21] | 2.3-4.5 (90%)[16, 22] |
| Flupentixol |  |  |  | 0.7-2.2 (90%)[13] |
| Fluphenazine |  |  |  | 1 (90%)[13] |
| Haloperidol |  |  |  | 0.8-31 (90%)[13] |
| Imipramine |  |  | 0.5-50 (40-90%)[1, 21, 23] | 1.7 (90%)[16-17] |
| Levomepromazine |  |  |  | 8-54 (90%)[13] |
| Milnacipran |  |  | 14 (90%)[24] | 63 (90%)[24] |
| Mirtazepine |  |  |  | 0.07 (90%)[16] |
| Nortriptyline |  |  | 2 (n/a)[3] |  |
| Paroxetine |  |  | 5 (90%)[21] |  |
| Reboxetine |  |  |  | 2.7 (90%)[16] |
| Risperidone |  |  |  | 100* (90%)[13] |
| Sertraline |  |  | 5 (90%)[21] |  |
| Spiperone |  |  | 8 (90%)[25-26] |  |
| Rhioridazine | 2600 (40%)[5] |  |  | 4.8 (90%)[13] |
| Trazodone |  |  | 10* (20%)[23] |  |
| Thioridazine | 2600 (40%)[5] |  |  | 4.8 (90%)[13] |
| Trifluoroperazine | 12 (90%)[5] | 100* (85%)[5] |  |  |
| Trimipramine |  |  |  | 8.3 (90%)[16] |

Table S2. Anesthetics (n=16)

| **Modulator** | **GABA_A_** | **GlyR** | **nAChR** | **5-HT3** |
| --- | --- | --- | --- | --- |
| Bupivacaine | 3000 (40%)[27] |  | 100 (90%)[27] | 50 (90%)[28] |
| Chloral hydrate | 1000-3900 (50-3500%)[29-30] | 3500-6000 (77-135%)[29-30] |  | 8000 (80%)[31] |
| Chloroform | Mixed[32] | 2000 (250-400%)[33-34] |  | (80%)[35] |
| Etomidate | 0.6-11 (28-169%)[29, 36] | Mixed[29, 34] | 35-57 (65-90%)[37-38] | 140-180 (90%)[39] |
| Halothane | Mixed[32] | 300 (70-400%)[32, 34] | 40-860 (90%)[40-41] | 400* (42%)[35] |
| Isoflurane | 300 (214%)[30] | 270 (172%)[30] | 67 (90%)[41] | (50%)[35] |
| Ketamine | 1200-700,000 (140-160%)[38, 42] |  | 0.24-17 (70-90%)[37-38, 43-45] | 10 (200%)[46] |
| Lidocaine | 1000 (30%)[47] | mixed[47] | 28 (90%)[48] | 8.5 (90%)[8, 49] |
| Methohexital | 1 (420%)[50] | 200* (20%)[34] |  | 100 (90%)[51] |
| Midazolam | 0.1-1.1 (92-342%)[52] |  | 140 (20%)[53] |  |
| NO2 | (20%)[54] | 200,000 (30-1500%)[34, 54] | (10-40%)[54] | (15%)[54] |
| Procaine | 1000 (60%)[47] | mixed[47] | 2.8 (90%)[48] | 3 (90%)[28] |
| Propofol | 2.3-3 (100-130%)[29, 36] | 27-50 (100-2000%)[29, 34] | 30-81 (80-90%)[37, 45] | 300-370 (90%)[39] |
| Tetracaine |  |  | 1-40 (90%)[55] | 30 (90%)[28] |
| Urethane | 50,000 (350%)[56] | 40,000 (350%)[56] | 100,000 (300%)[56] |  |
| Xenon | (20%)[54] | 2300 (30-50%)[34, 54] | (10-40%)[54] | (35%)[57] |

Table S3. Anticonvulsants (n=12)

| **Modulator** | **GABA_A_** | **GlyR** | **nAChR** | **5-HT3** |
| --- | --- | --- | --- | --- |
| Barbiturate^a^ | 20-70 (130-550%)[36, 58] | 800 (50-70%)[29, 34] | 32 (90%)[59] | 97-270 (90%)[39, 51] |
| Benzodiazepine | 0.05 (100%)[60] | 1* (15%)[61] | 66 (65%)[37] |  |
| Carbamazepine | 10* (20%)[61] | 10* (10%)[61] | 140 (85%)[62] |  |
| Ethosuximide | 1000* (10%)[61] | 1000* (25%)[61] |  |  |
| Felbamate | mixed[63] | 3000 (20%)[64] |  |  |
| Gabapentin | 150* (13%)[65] | 500 (10%)[64] |  |  |
| Levitiracetam | 30 (25%)[61] |  |  |  |
| Lamotrigine |  |  | 100 (65%)[66] |  |
| Phenytoin | Mixed[61, 67] | 50* (10%)[61] |  |  |
| Stiripentol | 30 (300%)[68] |  |  |  |
| Topirimate | 1.3-4.1mM (340-660%)[69] | 1000* (10-20%)[64, 70] |  |  |
| Valproic Acid | 1000* (25%)[61] | 1000* (20%)[61] |  |  |

a, includes pentobarbital and phenobarbital

Table S4. Extracts (n=17)

| **Modulator** | **GABA_A_** | **GlyR** | **nAChR** | **5-HT3** |
| --- | --- | --- | --- | --- |
| Apigenin | 8 (50%)[71-72] |  |  |  |
| Caffeine | 3.6-15mM (80%)[73-74] | 450 (90%)[74] |  |  |
| Camphor | 100 (40%)[75] |  |  |  |
| Catechin | 1700 (60%)[73] |  |  |  |
| Chrysin | 30* (12%)[72] |  |  |  |
| Coffee | mixed[76] |  |  |  |
| Cytisine |  |  | mixed[77] |  |
| Flavone | 30* (15%)[72] |  |  |  |
| Ginkgolides | 2-73 (50-90%)[78-79] | 0.3-3.7 (40-90%)[79-81] |  |  |
| Ginseng | 53 (X)[82] | 50 (300%)[83] | 17-106 (50-90%)[84-85] | 7-37 (23-52%)[86-88] |
| Menthol | 35 (500%)[75] | 35 (150%)[75] |  |  |
| Morine | 30* (38%)[72] |  |  |  |
| Quercetin | 5 (90%)[72] | 24 (85%)[89] | 30* (80%)[72] | 19 (80%)[72] |
| Thujone | 21-100 (25-60%)[75, 90] | n/a (45%)[75] |  | 60 (90%)[91] |
| Thymol | 20 (416%)[92] |  |  |  |
| Valerian | 2.5-18 (70-480%)[93] |  |  |  |
| Whiskey | variable (25-330%)[94] |  |  |  |

Table S5. Amino Acids and Ions (n=14)

| **Modulator** | **GABA_A_** | **GlyR** | **nAChR** | **5-HT3** |
| --- | --- | --- | --- | --- |
| Acetylcholine |  |  | Ag |  |
| β-alanine | Ag | Ag |  |  |
| Choline |  |  | Ag[95-96] |  |
| Dopamine |  |  |  | Ag[97-101] |
| GABA | Ag | 1000* (35%)[102] |  |  |
| Glycine | 20,000 (20%)[102] | Ag |  |  |
| Histamine | 1000 (100%)[103] |  |  |  |
| Proline |  | Ag[104] |  |  |
| Serotonin |  |  | 56-250 (75-90%)[25-26, 105-106] | Ag |
| Taurine | Ag | Ag |  |  |
| ---------------------------- |  |  |  |  |
| Ammonia | 200 (20%)[107] | 6000 (35%)[102, 108] |  |  |
| Copper | 9-1900 (90%)[109] | 4 (90%)[110] |  | 25 (>90%)[111] |
| Magnesium | 100 (50-75%)[112] |  |  | 1100 (60%)[113] |
| Zinc | 1-640 (90%)[114] | mixed[115] | mixed[116-117] | mixed[113, 118] |

Table S6. Steroids (n=11)

| **Modulator** | **GABA_A_** | **GlyR** | **nAChR** | **5-HT3** |
| --- | --- | --- | --- | --- |
| 17B-estradiol | n/a (20%)[119] |  | 5 (30%)[120] | 7 (90%)[121-122] |
| Allopregnanolone | 1* (225%)[123] | 20* (33%)[123] | 12 (15%)[120] | 10* (35%)[121] |
| Cortisol | Mixed[124] |  |  |  |
| Dexamethasone |  |  | 10 (90%)[125] | 5.3 (90%)[126] |
| DHEAS | 10 (90%)[119] | 4-46 (90%)[127] | 7 (25%)[120] |  |
| Hydrocortisone | 5-1000 (54%)[128] | 10* (X)[129] | 150 (90%)[130-131] |  |
| Methylprednisolone |  |  |  | 1.1 (90%)[126] |
| Pregnanolone sulfate | 7 (90%)[119] | 2-19 (90%)[127] |  |  |
| Progesterone | 26 (150%)[132] | 16-20 (20-60%)[132-133] | 3-9 (50-80%)[120, 134] | 31 (75%)[121, 135] |
| Testosterone | 78 (82%)[119] |  | 46 (50%)[134] | 10* (45%)[121] |
| THDOC | 0.1-1 (100-1500%)[136-137] | 12 (50%)[123] |  |  |

Table S7. Endogenous substances (n=15)

| **Modulator** | **GABA_A_** | **GlyR** | **nAChR** | **5-HT3** |
| --- | --- | --- | --- | --- |
| 5-hydroxyindole |  |  | 2500 (1200%)[138] | 100-1000 (20-30%)[97, 139-140] |
| Acetone | 50mM (40%)[141] | 500 (500%)[141] |  |  |
| Aldosterone |  |  | 100* (25%)[130] |  |
| β-amyloid |  |  | Mixed[142-144] |  |
| β-hydroxybuyrate | 10mM (15%)[141] | 10mM (350%)[141] |  |  |
| Dynorphin |  |  | 0.5 (90%)[145] |  |
| Hypoxanthine | 1000* (15%)[146] |  |  |  |
| Inosine | 1000* (12%)[146] |  |  |  |
| Kyneurenic acid |  |  | 7 (90%)[147] |  |
| Melatonin | mixed[148-149] | mixed[149] | <nM (70%)[150] |  |
| Methylisoguavosine | 500* (20%)[146] |  |  |  |
| Oleamide | 29 (216%)[151-152] | 22 (171%)[151] |  |  |
| Spermine | 100* (n/a)[153] |  | 20 (90%)[154] |  |
| Substance P |  |  | 2-50 (30%)[155] |  |
| Triiodothyronine | 7.3 (40%)[156] |  |  |  |

Table S8. Drugs of Abuse (n=5)

| **Modulator** | **GABA_A_** | **GlyR** | **nAChR** | **5-HT3** |
| --- | --- | --- | --- | --- |
| Cannabinoid |  | 0.07-0.32 (80%)[157] | 0.23 (90%)[158] | 0.1-0.3 (90%)[159-160] |
| Cocaine | 1100 (80%)[161] |  | 50 (80%)[162] | 0.1-0.7 (90%)[49, 163] |
| Alcohol | Variable[164] | 100mM (120%)[165-166] | Mixed | 50-300mM (15-190%) |
| Nicotine |  |  | Ag | 20 (n/a)[167] |
| PCP |  |  | 17 (90%)[2] |  |

Table S9. Miscellaneous Medications (n=53)

| **Modulator** | **GABA_A_** | **GlyR** | **nAChR** | **5-HT3** |
| --- | --- | --- | --- | --- |
| Amantadine |  |  | 3.4 (90%)[168] | 20-30 (n/a)[169] |
| Amiloride | 23-300 (90%)[170] |  |  |  |
| Aminophylline | Ag (~100s)[171] |  |  |  |
| Aspirin | 600 (20%)[172] |  |  |  |
| Atropine |  | 7-161 (90%)[173] | mixed[174] |  |
| Bemesetron |  |  |  | 0.001 (90%)[163] |
| Chloroquine | 460-670 (90%)[175] |  | 5 (90%)[176] | 24 (90%)[175] |
| Codeine |  |  | 100*, Ag[177] |  |
| Colchicine | 56 (90%)[178-179] | 64-324 (60-90%)[180] |  |  |
| Dextromethorphan |  | 3.3 (90%)[181] | 0.7-3.9 (90%)[182] |  |
| Diltiazem |  |  | 0.7 (70%)[183] | 5 (90%)[139, 184] |
| Dolasetron |  |  | 7.8 (90%)[185] | 0.01 (90%)[185] |
| Droperidol | 0.01 (25%)[186] |  | 5.8 (90%)[186] |  |
| Ergotamine |  |  |  | 30* (20%)[187] |
| Erythromycin |  |  | 80 (90%)[188] |  |
| Famotidine | 10-50 (60-80%)[189] |  |  |  |
| Fentanyl |  |  | 26 (65%)[37] |  |
| Furosemide | 10 (90%)[190] | 1000 (70%)[191] |  |  |
| Galantamine |  |  | mixed[192-193] |  |
| Genistein | 30-100 (50%)[71, 194] | 100* (30%)[194] |  |  |
| Gentamycin |  |  | 800 (75%)[188] |  |
| Granisetron |  |  | 4.4 (90%)[185] | <nM (90%)[185] |
| Hydrodolansetron |  |  | 0.8 (90%)[185] | <nM (90%)[185] |
| Ivermectin | 1* (80-400%)[195] | mixed[195-196] | 25 (300%)[197] |  |
| Mecamyline |  |  | 1* (50%)[2] |  |
| Mefloquine | 380-98,000 (90%)[175] |  |  | 0.7-2.7 (90%)[175] |
| Memantine |  |  | 0.4-16 (90%)[168, 198-199] | 3-30 (60-90%)[169, 200] |
| Meperidine |  |  | 16 (80%)[37] |  |
| Methadone |  |  | 35, Ag[201] |  |
| Methysergide |  |  | 22 (90%)[25-26] |  |
| Metochlopromide |  |  |  | 0.01-0.06 (90%)[113, 163, 187] |
| Morphine |  | 30 (90%)[202] | 30 (90%)[203] |  |
| Naloxone |  |  | 40 (90%)[203] |  |
| Naltrexone | 300* (35%)[204] | 300* (15%)[204] | 25-141 (90%)[204] |  |
| Nicardipine | 5* (30-50%)[205] | Mixed[205] |  |  |
| Nifedipine | 79 (90%)[206] | 1 (90%)[205] |  |  |
| Nimodipine |  |  | 10* (36%)[207] | 7 (desens)[184] |
| NSAIDs | mixed[208-209] |  |  |  |
| Ondansetron | 7 (90%)[210] | 25 (90%)[210] | 14-80 (90%)[185, 211] | <nM (90%)[113, 163, 185] |
| Penicillin | 560 (90%)[212] | 200 (80%)[213] | 700 (75%)[188] |  |
| Pentoxifylline | 200 (n/a)[74] |  |  |  |
| Physostigmine |  |  | Mixed[214] |  |
| Promethazine |  |  | 3 (50%)[2] |  |
| Quinine | 400-1700 (90%)[175] |  | 10 (90%)[176] | 1-16 (90%)[175] |
| Quinolones | 17-280 (70-90%)[215] | 100* (20%)[215] | 100* (10%)[215] |  |
| Ranitidine | 10* (12%)[189] |  |  |  |
| Riluzole | 59 (500%)[216-217] | 1000 (desens)[217] | 1000* (25%)[218] | 3.5 (65%)[98] |
| Tacrine |  |  | mixed[214] |  |
| Tamoxifen | 5* (230%)[219] | 5* (500%)[219] | 1.2 (90%)[220] | 0.8 (90%)[220] |
| Tetracycline |  |  | 30 (90%)[188] |  |
| Theophylline | 500-1840 (70-80%)[76, 221] | 390 (n/a)[74] |  |  |
| Tramodol | 50 (20%)[222] |  |  |  |
| Tropisetron |  | 5.4-84 (90%)[173] | mixed[211] | 0.001(90%)[163] |
| Varenicline |  |  | 2-55, Ag[223] |  |
| Verapamil | 193 (90%)[206] | 50* (70%)[205] |  | 4 (desens)[184] |

References

1. Gumilar F, Arias HR, Spitzmaul G, Bouzat C: **Molecular mechanisms of inhibition of nicotinic acetylcholine receptors by tricyclic antidepressants**. *Neuropharmacology* 2003, **45**(7):964-976.

2. Connolly J, Boulter J, Heinemann SF: **Alpha 4-2 beta 2 and other nicotinic acetylcholine receptor subtypes as targets of psychoactive and addictive drugs**. *Br J Pharmacol* 1992, **105**(3):657-666.

3. Schofield GG, Witkop B, Warnick JE, Albuquerque EX: **Differentiation of the open and closed states of the ionic channels of nicotinic acetylcholine receptors by tricyclic antidepressants**. *Proc Natl Acad Sci U S A* 1981, **78**(8):5240-5244.

4. Slemmer JE, Martin BR, Damaj MI: **Bupropion is a nicotinic antagonist**. *J Pharmacol Exp Ther* 2000, **295**(1):321-327.

5. Zorumski CF, Yang J: **Non-competitive inhibition of GABA currents by phenothiazines in cultured chick spinal cord and rat hippocampal neurons**. *Neurosci Lett* 1988, **92**(1):86-91.

6. Mozrzymas JW, Barberis A, Michalak K, Cherubini E: **Chlorpromazine inhibits miniature GABAergic currents by reducing the binding and by increasing the unbinding rate of GABAA receptors**. *J Neurosci* 1999, **19**(7):2474-2488.

7. Benoit P, Changeux JP: **Voltage dependencies of the effects of chlorpromazine on the nicotinic receptor channel from mouse muscle cell line So18**. *Neurosci Lett* 1993, **160**(1):81-84.

8. Sepulveda MI, Baker J, Lummis SC: **Chlorpromazine and QX222 block 5-HT3 receptors in N1E-115 neuroblastoma cells**. *Neuropharmacology* 1994, **33**(3-4):493-499.

9. Lopez-Valdes HE, Garcia-Colunga J, Miledi R: **Effects of clomipramine on neuronal nicotinic acetylcholine receptors**. *Eur J Pharmacol* 2002, **444**(1-2):13-19.

10. Michel FJ, Trudeau LE: **Clozapine inhibits synaptic transmission at GABAergic synapses established by ventral tegmental area neurones in culture**. *Neuropharmacology* 2000, **39**(9):1536-1543.

11. Nguyen QT, Miledi R: **Inhibition of skeletal muscle nicotinic receptors by the atypical antipsychotic clozapine**. *Neuropharmacology* 2002, **42**(5):662-669.

12. Singhal SK, Zhang L, Morales M, Oz M: **Antipsychotic clozapine inhibits the function of alpha7-nicotinic acetylcholine receptors**. *Neuropharmacology* 2007, **52**(2):387-394.

13. Rammes G, Eisensamer B, Ferrari U, Shapa M, Gimpl G, Gilling K, Parsons C, Riering K, Hapfelmeier G, Bondy B *et al*: **Antipsychotic drugs antagonize human serotonin type 3 receptor currents in a noncompetitive manner**. *Mol Psychiatry* 2004, **9**(9):846-858, 818.

14. Hermann B, Wetzel CH, Pestel E, Zieglgansberger W, Holsboer F, Rupprecht R: **Functional antagonistic properties of clozapine at the 5-HT3 receptor**. *Biochem Biophys Res Commun* 1996, **225**(3):957-960.

15. Izaguirre V, Fernandez-Fernandez JM, Cena V, Gonzalez-Garcia C: **Tricyclic antidepressants block cholinergic nicotinic receptors and ATP secretion in bovine chromaffin cells**. *FEBS Lett* 1997, **418**(1-2):39-42.

16. Eisensamer B, Rammes G, Gimpl G, Shapa M, Ferrari U, Hapfelmeier G, Bondy B, Parsons C, Gilling K, Zieglgansberger W *et al*: **Antidepressants are functional antagonists at the serotonin type 3 (5-HT3) receptor**. *Mol Psychiatry* 2003, **8**(12):994-1007.

17. Gumilar F, Bouzat C: **Tricyclic antidepressants inhibit homomeric Cys-loop receptors by acting at different conformational states**. *Eur J Pharmacol* 2008, **584**(1):30-39.

18. Robinson RT, Drafts BC, Fisher JL: **Fluoxetine increases GABA(A) receptor activity through a novel modulatory site**. *J Pharmacol Exp Ther* 2003, **304**(3):978-984.

19. Maggi L, Palma E, Miledi R, Eusebi F: **Effects of fluoxetine on wild and mutant neuronal alpha 7 nicotinic receptors**. *Mol Psychiatry* 1998, **3**(4):350-355.

20. Garcia-Colunga J, Awad JN, Miledi R: **Blockage of muscle and neuronal nicotinic acetylcholine receptors by fluoxetine (Prozac)**. *Proc Natl Acad Sci U S A* 1997, **94**(5):2041-2044.

21. Feuerbach D, Lingenhohl K, Dobbins P, Mosbacher J, Corbett N, Nozulak J, Hoyer D: **Coupling of human nicotinic acetylcholine receptors alpha 7 to calcium channels in GH3 cells**. *Neuropharmacology* 2005, **48**(2):215-227.

22. Choi JS, Choi BH, Ahn HS, Kim MJ, Rhie DJ, Yoon SH, Min DS, Jo YH, Kim MS, Sung KW *et al*: **Mechanism of block by fluoxetine of 5-hydroxytryptamine3 (5-HT3)-mediated currents in NCB-20 neuroblastoma cells**. *Biochem Pharmacol* 2003, **66**(11):2125-2132.

23. Lopez-Valdes HE, Garcia-Colunga J: **Antagonism of nicotinic acetylcholine receptors by inhibitors of monoamine uptake**. *Mol Psychiatry* 2001, **6**(5):511-519.

24. Ueta K, Suzuki T, Uchida I, Mashimo T: **In vitro inhibition of recombinant ligand-gated ion channels by high concentrations of milnacipran**. *Psychopharmacology (Berl)* 2004, **175**(2):241-246.

25. Garcia-Colunga J, Miledi R: **Blockage of mouse muscle nicotinic receptors by serotonergic compounds**. *Exp Physiol* 1999, **84**(5):847-864.

26. Garcia-Colunga J, Miledi R: **Effects of serotonergic agents on neuronal nicotinic acetylcholine receptors**. *Proc Natl Acad Sci U S A* 1995, **92**(7):2919-2923.

27. Ueta K, Sugimoto M, Suzuki T, Uchida I, Mashimo T: **In vitro antagonism of recombinant ligand-gated ion-channel receptors by stereospecific enantiomers of bupivacaine**. *Reg Anesth Pain Med* 2006, **31**(1):19-25.

28. Ueta K, Suzuki T, Sugimoto M, Uchida I, Mashimo T: **Local anesthetics have different mechanisms and sites of action at recombinant 5-HT3 receptors**. *Reg Anesth Pain Med* 2007, **32**(6):462-470.

29. Pistis M, Belelli D, Peters JA, Lambert JJ: **The interaction of general anaesthetics with recombinant GABAA and glycine receptors expressed in Xenopus laevis oocytes: a comparative study**. *Br J Pharmacol* 1997, **122**(8):1707-1719.

30. Krasowski MD, Harrison NL: **The actions of ether, alcohol and alkane general anaesthetics on GABAA and glycine receptors and the effects of TM2 and TM3 mutations**. *Br J Pharmacol* 2000, **129**(4):731-743.

31. Downie DL, Hope AG, Belelli D, Lambert JJ, Peters JA, Bentley KR, Steward LJ, Chen CY, Barnes NM: **The interaction of trichloroethanol with murine recombinant 5-HT3 receptors**. *Br J Pharmacol* 1995, **114**(8):1641-1651.

32. Jenkins A, Lobo IA, Gong D, Trudell JR, Solt K, Harris RA, Eger EI, 2nd: **General anesthetics have additive actions on three ligand gated ion channels**. *Anesth Analg* 2008, **107**(2):486-493.

33. Beckstead MJ, Phelan R, Mihic SJ: **Antagonism of inhalant and volatile anesthetic enhancement of glycine receptor function**. *J Biol Chem* 2001, **276**(27):24959-24964.

34. Daniels S, Roberts RJ: **Post-synaptic inhibitory mechanisms of anaesthesia; glycine receptors**. *Toxicol Lett* 1998, **100-101**:71-76.

35. Stevens R, Rusch D, Solt K, Raines DE, Davies PA: **Modulation of human 5-hydroxytryptamine type 3AB receptors by volatile anesthetics and n-alcohols**. *J Pharmacol Exp Ther* 2005, **314**(1):338-345.

36. Hill-Venning C, Belelli D, Peters JA, Lambert JJ: **Subunit-dependent interaction of the general anaesthetic etomidate with the gamma-aminobutyric acid type A receptor**. *Br J Pharmacol* 1997, **120**(5):749-756.

37. Wachtel RE, Wegrzynowicz ES: **Kinetics of nicotinic acetylcholine ion channels in the presence of intravenous anaesthetics and induction agents**. *Br J Pharmacol* 1992, **106**(3):623-627.

38. Flood P, Krasowski MD: **Intravenous anesthetics differentially modulate ligand-gated ion channels**. *Anesthesiology* 2000, **92**(5):1418-1425.

39. Rusch D, Braun HA, Wulf H, Schuster A, Raines DE: **Inhibition of human 5-HT(3A) and 5-HT(3AB) receptors by etomidate, propofol and pentobarbital**. *Eur J Pharmacol* 2007, **573**(1-3):60-64.

40. Downie DL, Vicente-Agullo F, Campos-Caro A, Bushell TJ, Lieb WR, Franks NP: **Determinants of the anesthetic sensitivity of neuronal nicotinic acetylcholine receptors**. *J Biol Chem* 2002, **277**(12):10367-10373.

41. Yamashita M, Mori T, Nagata K, Yeh JZ, Narahashi T: **Isoflurane modulation of neuronal nicotinic acetylcholine receptors expressed in human embryonic kidney cells**. *Anesthesiology* 2005, **102**(1):76-84.

42. Lin LH, Chen LL, Zirrolli JA, Harris RA: **General anesthetics potentiate gamma-aminobutyric acid actions on gamma-aminobutyric acidA receptors expressed by Xenopus oocytes: lack of involvement of intracellular calcium**. *J Pharmacol Exp Ther* 1992, **263**(2):569-578.

43. Ho KK, Flood P: **Single amino acid residue in the extracellular portion of transmembrane segment 2 in the nicotinic alpha7 acetylcholine receptor modulates sensitivity to ketamine**. *Anesthesiology* 2004, **100**(3):657-662.

44. Scheller M, Bufler J, Hertle I, Schneck HJ, Franke C, Kochs E: **Ketamine blocks currents through mammalian nicotinic acetylcholine receptor channels by interaction with both the open and the closed state**. *Anesth Analg* 1996, **83**(4):830-836.

45. Furuya R, Oka K, Watanabe I, Kamiya Y, Itoh H, Andoh T: **The effects of ketamine and propofol on neuronal nicotinic acetylcholine receptors and P2x purinoceptors in PC12 cells**. *Anesth Analg* 1999, **88**(1):174-180.

46. Peters JA, Malone HM, Lambert JJ: **Ketamine potentiates 5-HT3 receptor-mediated currents in rabbit nodose ganglion neurones**. *Br J Pharmacol* 1991, **103**(3):1623-1625.

47. Hara K, Sata T: **The effects of the local anesthetics lidocaine and procaine on glycine and gamma-aminobutyric acid receptors expressed in Xenopus oocytes**. *Anesth Analg* 2007, **104**(6):1434-1439, table of contents.

48. Cuevas J, Adams DJ: **Local anaesthetic blockade of neuronal nicotinic ACh receptor-channels in rat parasympathetic ganglion cells**. *Br J Pharmacol* 1994, **111**(3):663-672.

49. Fan P, Oz M, Zhang L, Weight FF: **Effect of cocaine on the 5-HT3 receptor-mediated ion current in Xenopus oocytes**. *Brain Res* 1995, **673**(2):181-184.

50. Koltchine VV, Ye Q, Finn SE, Harrison NL: **Chimeric GABAA/glycine receptors: expression and barbiturate pharmacology**. *Neuropharmacology* 1996, **35**(9-10):1445-1456.

51. Barann M, Meder W, Dorner Z, Bruss M, Bonisch H, Gothert M, Urban BW: **Recombinant human 5-HT3A receptors in outside-out patches of HEK 293 cells: basic properties and barbiturate effects**. *Naunyn Schmiedebergs Arch Pharmacol* 2000, **362**(3):255-265.

52. Khom S, Baburin I, Timin EN, Hohaus A, Sieghart W, Hering S: **Pharmacological properties of GABAA receptors containing gamma1 subunits**. *Mol Pharmacol* 2006, **69**(2):640-649.

53. Hertle I, Scheller M, Bufler J, Schneck HJ, Stocker M, Kochs E, Franke C: **Interaction of midazolam with the nicotinic acetylcholine receptor of mouse myotubes**. *Anesth Analg* 1997, **85**(1):174-181.

54. Yamakura T, Harris RA: **Effects of gaseous anesthetics nitrous oxide and xenon on ligand-gated ion channels. Comparison with isoflurane and ethanol**. *Anesthesiology* 2000, **93**(4):1095-1101.

55. Eterovic VA, Li L, Ferchmin PA, Lee YH, Hann RM, Rodriguez AD, McNamee MG: **The ion channel of muscle and electric organ acetylcholine receptors: differing affinities for noncompetitive inhibitors**. *Cell Mol Neurobiol* 1993, **13**(2):111-121.

56. Hara K, Harris RA: **The anesthetic mechanism of urethane: the effects on neurotransmitter-gated ion channels**. *Anesth Analg* 2002, **94**(2):313-318, table of contents.

57. Suzuki T, Koyama H, Sugimoto M, Uchida I, Mashimo T: **The diverse actions of volatile and gaseous anesthetics on human-cloned 5-hydroxytryptamine3 receptors expressed in Xenopus oocytes**. *Anesthesiology* 2002, **96**(3):699-704.

58. Thompson SA, Whiting PJ, Wafford KA: **Barbiturate interactions at the human GABAA receptor: dependence on receptor subunit combination**. *Br J Pharmacol* 1996, **117**(3):521-527.

59. Dilger JP, Boguslavsky R, Barann M, Katz T, Vidal AM: **Mechanisms of barbiturate inhibition of acetylcholine receptor channels**. *J Gen Physiol* 1997, **109**(3):401-414.

60. Wafford KA, Bain CJ, Whiting PJ, Kemp JA: **Functional comparison of the role of gamma subunits in recombinant human gamma-aminobutyric acidA/benzodiazepine receptors**. *Mol Pharmacol* 1993, **44**(2):437-442.

61. Rigo JM, Hans G, Nguyen L, Rocher V, Belachew S, Malgrange B, Leprince P, Moonen G, Selak I, Matagne A *et al*: **The anti-epileptic drug levetiracetam reverses the inhibition by negative allosteric modulators of neuronal GABA- and glycine-gated currents**. *Br J Pharmacol* 2002, **136**(5):659-672.

62. Picard F, Bertrand S, Steinlein OK, Bertrand D: **Mutated nicotinic receptors responsible for autosomal dominant nocturnal frontal lobe epilepsy are more sensitive to carbamazepine**. *Epilepsia* 1999, **40**(9):1198-1209.

63. Simeone TA, Otto JF, Wilcox KS, White HS: **Felbamate is a subunit selective modulator of recombinant gamma-aminobutyric acid type A receptors expressed in Xenopus oocytes**. *Eur J Pharmacol* 2006, **552**(1-3):31-35.

64. Karkar KM, Thio LL, Yamada KA: **Effects of seven clinically important antiepileptic drugs on inhibitory glycine receptor currents in hippocampal neurons**. *Epilepsy Res* 2004, **58**(1):27-35.

65. Stefani A, Spadoni F, Giacomini P, Lavaroni F, Bernardi G: **The effects of gabapentin on different ligand- and voltage-gated currents in isolated cortical neurons**. *Epilepsy Res* 2001, **43**(3):239-248.

66. Valles AS, Garbus I, Barrantes FJ: **Lamotrigine is an open-channel blocker of the nicotinic acetylcholine receptor**. *Neuroreport* 2007, **18**(1):45-50.

67. McLean MJ, Macdonald RL: **Multiple actions of phenytoin on mouse spinal cord neurons in cell culture**. *J Pharmacol Exp Ther* 1983, **227**(3):779-789.

68. Fisher JL: **The anti-convulsant stiripentol acts directly on the GABA(A) receptor as a positive allosteric modulator**. *Neuropharmacology* 2008.

69. Simeone TA, Wilcox KS, White HS: **Subunit selectivity of topiramate modulation of heteromeric GABA(A) receptors**. *Neuropharmacology* 2006, **50**(7):845-857.

70. Mohammadi B, Krampfl K, Cetinkaya C, Wolfes H, Dengler R, Bufler J: **Interaction of topiramate with glycine receptor channels**. *Pharmacol Res* 2005, **51**(6):587-592.

71. Campbell EL, Chebib M, Johnston GA: **The dietary flavonoids apigenin and (-)-epigallocatechin gallate enhance the positive modulation by diazepam of the activation by GABA of recombinant GABA(A) receptors**. *Biochem Pharmacol* 2004, **68**(8):1631-1638.

72. Goutman JD, Waxemberg MD, Donate-Oliver F, Pomata PE, Calvo DJ: **Flavonoid modulation of ionic currents mediated by GABA(A) and GABA(C) receptors**. *Eur J Pharmacol* 2003, **461**(2-3):79-87.

73. Hossain SJ, Hamamoto K, Aoshima H, Hara Y: **Effects of tea components on the response of GABA(A) receptors expressed in Xenopus Oocytes**. *J Agric Food Chem* 2002, **50**(14):3954-3960.

74. Uneyama H, Harata N, Akaike N: **Caffeine and related compounds block inhibitory amino acid-gated Cl- currents in freshly dissociated rat hippocampal neurones**. *Br J Pharmacol* 1993, **109**(2):459-465.

75. Hall AC, Turcotte CM, Betts BA, Yeung WY, Agyeman AS, Burk LA: **Modulation of human GABAA and glycine receptor currents by menthol and related monoterpenoids**. *Eur J Pharmacol* 2004, **506**(1):9-16.

76. Hossain SJ, Aoshima H, Koda H, Kiso Y: **Effects of coffee components on the response of GABA(A) receptors expressed in Xenopus oocytes**. *J Agric Food Chem* 2003, **51**(26):7568-7575.

77. Houlihan LM, Slater Y, Guerra DL, Peng JH, Kuo YP, Lukas RJ, Cassels BK, Bermudez I: **Activity of cytisine and its brominated isosteres on recombinant human alpha7, alpha4beta2 and alpha4beta4 nicotinic acetylcholine receptors**. *J Neurochem* 2001, **78**(5):1029-1043.

78. Huang SH, Duke RK, Chebib M, Sasaki K, Wada K, Johnston GA: **Ginkgolides, diterpene trilactones of Ginkgo biloba, as antagonists at recombinant alpha1beta2gamma2L GABAA receptors**. *Eur J Pharmacol* 2004, **494**(2-3):131-138.

79. Ivic L, Sands TT, Fishkin N, Nakanishi K, Kriegstein AR, Stromgaard K: **Terpene trilactones from Ginkgo biloba are antagonists of cortical glycine and GABA(A) receptors**. *J Biol Chem* 2003, **278**(49):49279-49285.

80. Chatterjee SS, Kondratskaya EL, Krishtal OA: **Structure-activity studies with Ginkgo biloba extract constituents as receptor-gated chloride channel blockers and modulators**. *Pharmacopsychiatry* 2003, **36 Suppl 1**:S68-77.

81. Kondratskaya EL, Betz H, Krishtal OA, Laube B: **The beta subunit increases the ginkgolide B sensitivity of inhibitory glycine receptors**. *Neuropharmacology* 2005, **49**(6):945-951.

82. Choi SE, Choi S, Lee JH, Whiting PJ, Lee SM, Nah SY: **Effects of ginsenosides on GABA(A) receptor channels expressed in Xenopus oocytes**. *Arch Pharm Res* 2003, **26**(1):28-33.

83. Noh JH, Choi S, Lee JH, Betz H, Kim JI, Park CS, Lee SM, Nah SY: **Effects of ginsenosides on glycine receptor alpha1 channels expressed in Xenopus oocytes**. *Mol Cells* 2003, **15**(1):34-39.

84. Choi S, Jung SY, Lee JH, Sala F, Criado M, Mulet J, Valor LM, Sala S, Engel AG, Nah SY: **Effects of ginsenosides, active components of ginseng, on nicotinic acetylcholine receptors expressed in Xenopus oocytes**. *Eur J Pharmacol* 2002, **442**(1-2):37-45.

85. Sala F, Mulet J, Choi S, Jung SY, Nah SY, Rhim H, Valor LM, Criado M, Sala S: **Effects of ginsenoside Rg2 on human neuronal nicotinic acetylcholine receptors**. *J Pharmacol Exp Ther* 2002, **301**(3):1052-1059.

86. Choi S, Lee JH, Oh S, Rhim H, Lee SM, Nah SY: **Effects of ginsenoside Rg2 on the 5-HT3A receptor-mediated ion current in Xenopus oocytes**. *Mol Cells* 2003, **15**(1):108-113.

87. Lee BH, Jeong SM, Lee JH, Kim DH, Kim JH, Kim JI, Shin HC, Lee SM, Nah SY: **Differential effect of ginsenoside metabolites on the 5-HT3A receptor-mediated ion current in Xenopus oocytes**. *Mol Cells* 2004, **17**(1):51-56.

88. Min KT, Koo BN, Kang JW, Bai SJ, Ko SR, Cho ZH: **Effect of ginseng saponins on the recombinant serotonin type 3A receptor expressed in xenopus oocytes: implication of possible application as an antiemetic**. *J Altern Complement Med* 2003, **9**(4):505-510.

89. Lee BH, Pyo MK, Lee JH, Choi SH, Shin TJ, Lee SM, Lim Y, Han YS, Paik HD, Cho SG *et al*: **Differential regulations of quercetin and its glycosides on ligand-gated ion channels**. *Biol Pharm Bull* 2008, **31**(4):611-617.

90. Hold KM, Sirisoma NS, Ikeda T, Narahashi T, Casida JE: **Alpha-thujone (the active component of absinthe): gamma-aminobutyric acid type A receptor modulation and metabolic detoxification**. *Proc Natl Acad Sci U S A* 2000, **97**(8):3826-3831.

91. Deiml T, Haseneder R, Zieglgansberger W, Rammes G, Eisensamer B, Rupprecht R, Hapfelmeier G: **Alpha-thujone reduces 5-HT3 receptor activity by an effect on the agonist-reduced desensitization**. *Neuropharmacology* 2004, **46**(2):192-201.

92. Priestley CM, Williamson EM, Wafford KA, Sattelle DB: **Thymol, a constituent of thyme essential oil, is a positive allosteric modulator of human GABA(A) receptors and a homo-oligomeric GABA receptor from Drosophila melanogaster**. *Br J Pharmacol* 2003, **140**(8):1363-1372.

93. Khom S, Baburin I, Timin E, Hohaus A, Trauner G, Kopp B, Hering S: **Valerenic acid potentiates and inhibits GABA(A) receptors: molecular mechanism and subunit specificity**. *Neuropharmacology* 2007, **53**(1):178-187.

94. Hossain SJ, Aoshima H, Koda H, Kiso Y: **Potentiation of the ionotropic GABA receptor response by whiskey fragrance**. *J Agric Food Chem* 2002, **50**(23):6828-6834.

95. Zwart R, Vijverberg HP: **Potentiation and inhibition of neuronal alpha4beta4 nicotinic acetylcholine receptors by choline**. *Eur J Pharmacol* 2000, **393**(1-3):209-214.

96. Zhao L, Kuo YP, George AA, Peng JH, Purandare MS, Schroeder KM, Lukas RJ, Wu J: **Functional properties of homomeric, human alpha 7-nicotinic acetylcholine receptors heterologously expressed in the SH-EP1 human epithelial cell line**. *J Pharmacol Exp Ther* 2003, **305**(3):1132-1141.

97. Hu XQ, Lovinger DM: **The L293 residue in transmembrane domain 2 of the 5-HT3A receptor is a molecular determinant of allosteric modulation by 5-hydroxyindole**. *Neuropharmacology* 2008, **54**(8):1153-1165.

98. Kim KJ, Cho HS, Choi SJ, Jeun SH, Kim SY, Sung KW: **Direct effects of riluzole on 5-hydroxytryptamine (5-HT)3 receptor-activated ion currents in NCB-20 neuroblastoma cells**. *J Pharmacol Sci* 2008, **107**(1):57-65.

99. Solt K, Ruesch D, Forman SA, Davies PA, Raines DE: **Differential effects of serotonin and dopamine on human 5-HT3A receptor kinetics: interpretation within an allosteric kinetic model**. *J Neurosci* 2007, **27**(48):13151-13160.

100. Solt K, Stevens RJ, Davies PA, Raines DE: **General anesthetic-induced channel gating enhancement of 5-hydroxytryptamine type 3 receptors depends on receptor subunit composition**. *J Pharmacol Exp Ther* 2005, **315**(2):771-776.

101. Oz M, Zhang L, Rotondo A, Sun H, Morales M: **Direct activation by dopamine of recombinant human 5-HT1A receptors: comparison with human 5-HT2C and 5-HT3 receptors**. *Synapse* 2003, **50**(4):303-313.

102. Yang L, Sonner JM: **The anesthetic-like effects of diverse compounds on wild-type and mutant gamma-aminobutyric acid type A and glycine receptors**. *Anesth Analg* 2008, **106**(3):838-845, table of contents.

103. Saras A, Gisselmann G, Vogt-Eisele AK, Erlkamp KS, Kletke O, Pusch H, Hatt H: **Histamine action on vertebrate GABAA receptors: direct channel gating and potentiation of GABA responses**. *J Biol Chem* 2008, **283**(16):10470-10475.

104. Henzi V, Reichling DB, Helm SW, MacDermott AB: **L-proline activates glutamate and glycine receptors in cultured rat dorsal horn neurons**. *Mol Pharmacol* 1992, **41**(4):793-801.

105. Rothlin CV, Katz E, Verbitsky M, Elgoyhen AB: **The alpha9 nicotinic acetylcholine receptor shares pharmacological properties with type A gamma-aminobutyric acid, glycine, and type 3 serotonin receptors**. *Mol Pharmacol* 1999, **55**(2):248-254.

106. Fucile S, Palma E, Eusebi F, Miledi R: **Serotonin antagonizes the human neuronal alpha7 nicotinic acetylcholine receptor and becomes an agonist after L248T alpha7 mutation**. *Neuroscience* 2002, **110**(1):169-179.

107. Takahashi K, Kameda H, Kataoka M, Sanjou K, Harata N, Akaike N: **Ammonia potentiates GABAA response in dissociated rat cortical neurons**. *Neurosci Lett* 1993, **151**(1):51-54.

108. Brosnan RJ, Yang L, Milutinovic PS, Zhao J, Laster MJ, Eger EI, 2nd, Sonner JM: **Ammonia has anesthetic properties**. *Anesth Analg* 2007, **104**(6):1430-1433, table of contents.

109. Fisher JL, Macdonald RL: **The role of an alpha subtype M2-M3 His in regulating inhibition of GABAA receptor current by zinc and other divalent cations**. *J Neurosci* 1998, **18**(8):2944-2953.

110. Chen Z, Dillon GH, Huang R: **Identification of residues critical for Cu2+-mediated inhibition of glycine alpha1 receptors**. *Neuropharmacology* 2006, **51**(4):701-708.

111. Lovinger DM: **Inhibition of 5-HT3 receptor-mediated ion current by divalent metal cations in NCB-20 neuroblastoma cells**. *J Neurophysiol* 1991, **66**(4):1329-1337.

112. Moykkynen T, Uusi-Oukari M, Heikkila J, Lovinger DM, Luddens H, Korpi ER: **Magnesium potentiation of the function of native and recombinant GABA(A) receptors**. *Neuroreport* 2001, **12**(10):2175-2179.

113. Gill CH, Peters JA, Lambert JJ: **An electrophysiological investigation of the properties of a murine recombinant 5-HT3 receptor stably expressed in HEK 293 cells**. *Br J Pharmacol* 1995, **114**(6):1211-1221.

114. Krishek BJ, Moss SJ, Smart TG: **Interaction of H+ and Zn2+ on recombinant and native rat neuronal GABAA receptors**. *J Physiol* 1998, **507 ( Pt 3)**:639-652.

115. Laube B, Kuhse J, Rundstrom N, Kirsch J, Schmieden V, Betz H: **Modulation by zinc ions of native rat and recombinant human inhibitory glycine receptors**. *J Physiol* 1995, **483 ( Pt 3)**:613-619.

116. Palma E, Maggi L, Miledi R, Eusebi F: **Effects of Zn2+ on wild and mutant neuronal alpha7 nicotinic receptors**. *Proc Natl Acad Sci U S A* 1998, **95**(17):10246-10250.

117. Hsiao B, Dweck D, Luetje CW: **Subunit-dependent modulation of neuronal nicotinic receptors by zinc**. *J Neurosci* 2001, **21**(6):1848-1856.

118. Hubbard PC, Lummis SC: **Zn(2+) enhancement of the recombinant 5-HT(3) receptor is modulated by divalent cations**. *Eur J Pharmacol* 2000, **394**(2-3):189-197.

119. Park-Chung M, Malayev A, Purdy RH, Gibbs TT, Farb DH: **Sulfated and unsulfated steroids modulate gamma-aminobutyric acidA receptor function through distinct sites**. *Brain Res* 1999, **830**(1):72-87.

120. Paradiso K, Sabey K, Evers AS, Zorumski CF, Covey DF, Steinbach JH: **Steroid inhibition of rat neuronal nicotinic alpha4beta2 receptors expressed in HEK 293 cells**. *Mol Pharmacol* 2000, **58**(2):341-351.

121. Wetzel CH, Hermann B, Behl C, Pestel E, Rammes G, Zieglgansberger W, Holsboer F, Rupprecht R: **Functional antagonism of gonadal steroids at the 5-hydroxytryptamine type 3 receptor**. *Mol Endocrinol* 1998, **12**(9):1441-1451.

122. Oz M, Zhang L, Spivak CE: **Direct noncompetitive inhibition of 5-HT(3) receptor-mediated responses by forskolin and steroids**. *Arch Biochem Biophys* 2002, **404**(2):293-301.

123. Ueno S, Tsutsui M, Toyohira Y, Minami K, Yanagihara N: **Sites of positive allosteric modulation by neurosteroids on ionotropic gamma-aminobutyric acid receptor subunits**. *FEBS Lett* 2004, **566**(1-3):213-217.

124. Ong J, Kerr DI, Johnston GA: **Cortisol: a potent biphasic modulator at GABAA-receptor complexes in the guinea pig isolated ileum**. *Neurosci Lett* 1987, **82**(1):101-106.

125. Inoue M, Kuriyama H: **Glucocorticoids inhibit acetylcholine-induced current in chromaffin cells**. *Am J Physiol* 1989, **257**(5 Pt 1):C906-912.

126. Suzuki T, Sugimoto M, Koyama H, Mashimo T, Uchida I: **Inhibitory effect of glucocorticoids on human-cloned 5-hydroxytryptamine3A receptor expressed in xenopus oocytes**. *Anesthesiology* 2004, **101**(3):660-665.

127. Fodor L, Boros A, Dezso P, Maksay G: **Expression of heteromeric glycine receptor-channels in rat spinal cultures and inhibition by neuroactive steroids**. *Neurochem Int* 2006, **49**(6):577-583.

128. Ariyoshi M, Akasu T: **Voltage-clamp studies of the inhibition of gamma-aminobutyric acid response by glucocorticoids in bullfrog primary afferent neurons**. *Brain Res* 1987, **435**(1-2):241-248.

129. Prince RJ, Simmonds MA: **Steroid modulation of the strychnine-sensitive glycine receptor**. *Neuropharmacology* 1992, **31**(3):201-205.

130. Uki M, Nabekura J, Akaike N: **Suppression of the nicotinic acetylcholine response in rat superior cervical ganglionic neurons by steroids**. *J Neurochem* 1999, **72**(2):808-814.

131. Bouzat C, Barrantes FJ: **Modulation of muscle nicotinic acetylcholine receptors by the glucocorticoid hydrocortisone. Possible allosteric mechanism of channel blockade**. *J Biol Chem* 1996, **271**(42):25835-25841.

132. Wu FS, Gibbs TT, Farb DH: **Inverse modulation of gamma-aminobutyric acid- and glycine-induced currents by progesterone**. *Mol Pharmacol* 1990, **37**(5):597-602.

133. Maksay G, Laube B, Betz H: **Subunit-specific modulation of glycine receptors by neurosteroids**. *Neuropharmacology* 2001, **41**(3):369-376.

134. Valera S, Ballivet M, Bertrand D: **Progesterone modulates a neuronal nicotinic acetylcholine receptor**. *Proc Natl Acad Sci U S A* 1992, **89**(20):9949-9953.

135. Wu FS, Lai CP, Liu BC: **Non-competitive inhibition of 5-HT3 receptor-mediated currents by progesterone in rat nodose ganglion neurons**. *Neurosci Lett* 2000, **278**(1-2):37-40.

136. Wohlfarth KM, Bianchi MT, Macdonald RL: **Enhanced neurosteroid potentiation of ternary GABA(A) receptors containing the delta subunit**. *J Neurosci* 2002, **22**(5):1541-1549.

137. Puia G, Santi MR, Vicini S, Pritchett DB, Purdy RH, Paul SM, Seeburg PH, Costa E: **Neurosteroids act on recombinant human GABAA receptors**. *Neuron* 1990, **4**(5):759-765.

138. Zwart R, De Filippi G, Broad LM, McPhie GI, Pearson KH, Baldwinson T, Sher E: **5-Hydroxyindole potentiates human alpha 7 nicotinic receptor-mediated responses and enhances acetylcholine-induced glutamate release in cerebellar slices**. *Neuropharmacology* 2002, **43**(3):374-384.

139. Gunthorpe MJ, Lummis SC: **Diltiazem causes open channel block of recombinant 5-HT3 receptors**. *J Physiol* 1999, **519 Pt 3**:713-722.

140. van Hooft JA, van der Haar E, Vijverberg HP: **Allosteric potentiation of the 5-HT3 receptor-mediated ion current in N1E-115 neuroblastoma cells by 5-hydroxyindole and analogues**. *Neuropharmacology* 1997, **36**(4-5):649-653.

141. Yang L, Zhao J, Milutinovic PS, Brosnan RJ, Eger EI, 2nd, Sonner JM: **Anesthetic properties of the ketone bodies beta-hydroxybutyric acid and acetone**. *Anesth Analg* 2007, **105**(3):673-679.

142. Pym L, Kemp M, Raymond-Delpech V, Buckingham S, Boyd CA, Sattelle D: **Subtype-specific actions of beta-amyloid peptides on recombinant human neuronal nicotinic acetylcholine receptors (alpha7, alpha4beta2, alpha3beta4) expressed in Xenopus laevis oocytes**. *Br J Pharmacol* 2005, **146**(7):964-971.

143. Liu Q, Kawai H, Berg DK: **beta -Amyloid peptide blocks the response of alpha 7-containing nicotinic receptors on hippocampal neurons**. *Proc Natl Acad Sci U S A* 2001, **98**(8):4734-4739.

144. Lamb PW, Melton MA, Yakel JL: **Inhibition of neuronal nicotinic acetylcholine receptor channels expressed in Xenopus oocytes by beta-amyloid1-42 peptide**. *J Mol Neurosci* 2005, **27**(1):13-21.

145. Itoh H, Andoh T, Watanabe I, Sasaki T, Kamiya Y, Okumura F: **Dynorphins directly inhibit neuronal nicotinic acetylcholine receptors in PC12 cells**. *Eur J Neurosci* 2000, **12**(4):1253-1262.

146. Skerritt JH, Macdonald RL: **Benzodiazepine receptor ligand actions on GABA responses. Beta-carbolines, purines**. *Eur J Pharmacol* 1984, **101**(1-2):135-141.

147. Hilmas C, Pereira EF, Alkondon M, Rassoulpour A, Schwarcz R, Albuquerque EX: **The brain metabolite kynurenic acid inhibits alpha7 nicotinic receptor activity and increases non-alpha7 nicotinic receptor expression: physiopathological implications**. *J Neurosci* 2001, **21**(19):7463-7473.

148. Wu FS, Yang YC, Tsai JJ: **Melatonin potentiates the GABA(A) receptor-mediated current in cultured chick spinal cord neurons**. *Neurosci Lett* 1999, **260**(3):177-180.

149. Li GL, Li P, Yang XL: **Melatonin modulates gamma-aminobutyric acid(A) receptor-mediated currents on isolated carp retinal neurons**. *Neurosci Lett* 2001, **301**(1):49-53.

150. Lax P: **Melatonin inhibits nicotinic currents in cultured rat cerebellar granule neurons**. *J Pineal Res* 2008, **44**(1):70-77.

151. Coyne L, Lees G, Nicholson RA, Zheng J, Neufield KD: **The sleep hormone oleamide modulates inhibitory ionotropic receptors in mammalian CNS in vitro**. *Br J Pharmacol* 2002, **135**(8):1977-1987.

152. Lees G, Edwards MD, Hassoni AA, Ganellin CR, Galanakis D: **Modulation of GABA(A) receptors and inhibitory synaptic currents by the endogenous CNS sleep regulator cis-9,10-octadecenoamide (cOA)**. *Br J Pharmacol* 1998, **124**(5):873-882.

153. Brackley P, Goodnow R, Jr., Nakanishi K, Sudan HL, Usherwood PN: **Spermine and philanthotoxin potentiate excitatory amino acid responses of Xenopus oocytes injected with rat and chick brain RNA**. *Neurosci Lett* 1990, **114**(1):51-56.

154. Haghighi AP, Cooper E: **Neuronal nicotinic acetylcholine receptors are blocked by intracellular spermine in a voltage-dependent manner**. *J Neurosci* 1998, **18**(11):4050-4062.

155. Clapham DE, Neher E: **Substance P reduces acetylcholine-induced currents in isolated bovine chromaffin cells**. *J Physiol* 1984, **347**:255-277.

156. Chapell R, Martin J, Machu TK, Leidenheimer NJ: **Direct channel-gating and modulatory effects of triiodothyronine on recombinant GABA(A) receptors**. *Eur J Pharmacol* 1998, **349**(1):115-121.

157. Hejazi N, Zhou C, Oz M, Sun H, Ye JH, Zhang L: **Delta9-tetrahydrocannabinol and endogenous cannabinoid anandamide directly potentiate the function of glycine receptors**. *Mol Pharmacol* 2006, **69**(3):991-997.

158. Oz M, Ravindran A, Diaz-Ruiz O, Zhang L, Morales M: **The endogenous cannabinoid anandamide inhibits alpha7 nicotinic acetylcholine receptor-mediated responses in Xenopus oocytes**. *J Pharmacol Exp Ther* 2003, **306**(3):1003-1010.

159. Barann M, Molderings G, Bruss M, Bonisch H, Urban BW, Gothert M: **Direct inhibition by cannabinoids of human 5-HT3A receptors: probable involvement of an allosteric modulatory site**. *Br J Pharmacol* 2002, **137**(5):589-596.

160. Xiong W, Hosoi M, Koo BN, Zhang L: **Anandamide inhibition of 5-HT3A receptors varies with receptor density and desensitization**. *Mol Pharmacol* 2008, **73**(2):314-322.

161. Ye JH, Liu PL, Wu WH, McArdle JJ: **Cocaine depresses GABAA current of hippocampal neurons**. *Brain Res* 1997, **770**(1-2):169-175.

162. Krivoshein AV, Hess GP: **Mechanism-based approach to the successful prevention of cocaine inhibition of the neuronal (alpha 3 beta 4) nicotinic acetylcholine receptor**. *Biochemistry* 2004, **43**(2):481-489.

163. Peters JA, Malone HM, Lambert JJ: **An electrophysiological investigation of the properties of 5-HT3 receptors of rabbit nodose ganglion neurones in culture**. *Br J Pharmacol* 1993, **110**(2):665-676.

164. Olsen RW, Hanchar HJ, Meera P, Wallner M: **GABAA receptor subtypes: the "one glass of wine" receptors**. *Alcohol* 2007, **41**(3):201-209.

165. Mascia MP, Machu TK, Harris RA: **Enhancement of homomeric glycine receptor function by long-chain alcohols and anaesthetics**. *Br J Pharmacol* 1996, **119**(7):1331-1336.

166. Mascia MP, Mihic SJ, Valenzuela CF, Schofield PR, Harris RA: **A single amino acid determines differences in ethanol actions on strychnine-sensitive glycine receptors**. *Mol Pharmacol* 1996, **50**(2):402-406.

167. Breitinger HG, Geetha N, Hess GP: **Inhibition of the serotonin 5-HT3 receptor by nicotine, cocaine, and fluoxetine investigated by rapid chemical kinetic techniques**. *Biochemistry* 2001, **40**(28):8419-8429.

168. Buisson B, Bertrand D: **Open-channel blockers at the human alpha4beta2 neuronal nicotinic acetylcholine receptor**. *Mol Pharmacol* 1998, **53**(3):555-563.

169. Rammes G, Rupprecht R, Ferrari U, Zieglgansberger W, Parsons CG: **The N-methyl-D-aspartate receptor channel blockers memantine, MRZ 2/579 and other amino-alkyl-cyclohexanes antagonise 5-HT(3) receptor currents in cultured HEK-293 and N1E-115 cell systems in a non-competitive manner**. *Neurosci Lett* 2001, **306**(1-2):81-84.

170. Fisher JL: **Amiloride inhibition of gamma-aminobutyric acid(A) receptors depends upon the alpha subunit subtype**. *Mol Pharmacol* 2002, **61**(6):1322-1328.

171. Wang CT, Blankenship AG, Anishchenko A, Elstrott J, Fikhman M, Nakanishi S, Feller MB: **GABA(A) receptor-mediated signaling alters the structure of spontaneous activity in the developing retina**. *J Neurosci* 2007, **27**(34):9130-9140.

172. Gong N, Zhang M, Zhang XB, Chen L, Sun GC, Xu TL: **The aspirin metabolite salicylate enhances neuronal excitation in rat hippocampal CA1 area through reducing GABAergic inhibition**. *Neuropharmacology* 2008, **54**(2):454-463.

173. Maksay G, Laube B, Betz H: **Selective blocking effects of tropisetron and atropine on recombinant glycine receptors**. *J Neurochem* 1999, **73**(2):802-806.

174. Zwart R, Vijverberg HP: **Potentiation and inhibition of neuronal nicotinic receptors by atropine: competitive and noncompetitive effects**. *Mol Pharmacol* 1997, **52**(5):886-895.

175. Thompson AJ, Lummis SC: **Antimalarial drugs inhibit human 5-HT(3) and GABA(A) but not GABA(C) receptors**. *Br J Pharmacol* 2008, **153**(8):1686-1696.

176. Ballestero JA, Plazas PV, Kracun S, Gomez-Casati ME, Taranda J, Rothlin CV, Katz E, Millar NS, Elgoyhen AB: **Effects of quinine, quinidine, and chloroquine on alpha9alpha10 nicotinic cholinergic receptors**. *Mol Pharmacol* 2005, **68**(3):822-829.

177. Storch A, Schrattenholz A, Cooper JC, Abdel Ghani EM, Gutbrod O, Weber KH, Reinhardt S, Lobron C, Hermsen B, Soskic V *et al*: **Physostigmine, galanthamine and codeine act as 'noncompetitive nicotinic receptor agonists' on clonal rat pheochromocytoma cells**. *Eur J Pharmacol* 1995, **290**(3):207-219.

178. Bueno OF, Leidenheimer NJ: **Colchicine inhibits GABA(A) receptors independently of microtubule depolymerization**. *Neuropharmacology* 1998, **37**(3):383-390.

179. Weiner JL, Buhler AV, Whatley VJ, Harris RA, Dunwiddie TV: **Colchicine is a competitive antagonist at human recombinant gamma-aminobutyric acidA receptors**. *J Pharmacol Exp Ther* 1998, **284**(1):95-102.

180. Machu TK: **Colchicine competitively antagonizes glycine receptors expressed in Xenopus oocytes**. *Neuropharmacology* 1998, **37**(3):391-396.

181. Takahama K, Fukushima H, Isohama Y, Kai H, Miyata T: **Inhibition of glycine currents by dextromethorphan in neurones dissociated from the guinea-pig nucleus tractus solitarii**. *Br J Pharmacol* 1997, **120**(4):690-694.

182. Hernandez SC, Bertolino M, Xiao Y, Pringle KE, Caruso FS, Kellar KJ: **Dextromethorphan and its metabolite dextrorphan block alpha3beta4 neuronal nicotinic receptors**. *J Pharmacol Exp Ther* 2000, **293**(3):962-967.

183. Gandia L, Villarroya M, Sala F, Reig JA, Viniegra S, Quintanar JL, Garcia AG, Gutierrez LM: **Inhibition of nicotinic receptor-mediated responses in bovine chromaffin cells by diltiazem**. *Br J Pharmacol* 1996, **118**(5):1301-1307.

184. Hargreaves AC, Gunthorpe MJ, Taylor CW, Lummis SC: **Direct inhibition of 5-hydroxytryptamine3 receptors by antagonists of L-type Ca2+ channels**. *Mol Pharmacol* 1996, **50**(5):1284-1294.

185. Paul M, Callahan R, Au J, Kindler CH, Yost CS: **Antiemetics of the 5-hydroxytryptamine 3A antagonist class inhibit muscle nicotinic acetylcholine receptors**. *Anesth Analg* 2005, **101**(3):715-721, table of contents.

186. Flood P, Coates KM: **Droperidol inhibits GABA(A) and neuronal nicotinic receptor activation**. *Anesthesiology* 2002, **96**(4):987-993.

187. Walkembach J, Bruss M, Urban BW, Barann M: **Interactions of metoclopramide and ergotamine with human 5-HT(3A) receptors and human 5-HT reuptake carriers**. *Br J Pharmacol* 2005, **146**(4):543-552.

188. Schlesinger F, Krampfl K, Haeseler G, Dengler R, Bufler J: **Competitive and open channel block of recombinant nAChR channels by different antibiotics**. *Neuromuscul Disord* 2004, **14**(5):307-312.

189. Cannon KE, Fleck MW, Hough LB: **Effects of cimetidine-like drugs on recombinant GABAA receptors**. *Life Sci* 2004, **75**(21):2551-2558.

190. Korpi ER, Kuner T, Seeburg PH, Luddens H: **Selective antagonist for the cerebellar granule cell-specific gamma-aminobutyric acid type A receptor**. *Mol Pharmacol* 1995, **47**(2):283-289.

191. Kumamoto E, Murata Y: **Action of furosemide on GABA- and glycine currents in rat septal cholinergic neurons in culture**. *Brain Res* 1997, **776**(1-2):246-249.

192. Lopes C, Pereira EF, Wu HQ, Purushottamachar P, Njar V, Schwarcz R, Albuquerque EX: **Competitive antagonism between the nicotinic allosteric potentiating ligand galantamine and kynurenic acid at alpha7* nicotinic receptors**. *J Pharmacol Exp Ther* 2007, **322**(1):48-58.

193. Texido L, Ros E, Martin-Satue M, Lopez S, Aleu J, Marsal J, Solsona C: **Effect of galantamine on the human alpha7 neuronal nicotinic acetylcholine receptor, the Torpedo nicotinic acetylcholine receptor and spontaneous cholinergic synaptic activity**. *Br J Pharmacol* 2005, **145**(5):672-678.

194. Huang RQ, Fang MJ, Dillon GH: **The tyrosine kinase inhibitor genistein directly inhibits GABAA receptors**. *Brain Res Mol Brain Res* 1999, **67**(1):177-183.

195. Dawson GR, Wafford KA, Smith A, Marshall GR, Bayley PJ, Schaeffer JM, Meinke PT, McKernan RM: **Anticonvulsant and adverse effects of avermectin analogs in mice are mediated through the gamma-aminobutyric acid(A) receptor**. *J Pharmacol Exp Ther* 2000, **295**(3):1051-1060.

196. Shan Q, Haddrill JL, Lynch JW: **Ivermectin, an unconventional agonist of the glycine receptor chloride channel**. *J Biol Chem* 2001, **276**(16):12556-12564.

197. Krause RM, Buisson B, Bertrand S, Corringer PJ, Galzi JL, Changeux JP, Bertrand D: **Ivermectin: a positive allosteric effector of the alpha7 neuronal nicotinic acetylcholine receptor**. *Mol Pharmacol* 1998, **53**(2):283-294.

198. Aracava Y, Pereira EF, Maelicke A, Albuquerque EX: **Memantine blocks alpha7* nicotinic acetylcholine receptors more potently than n-methyl-D-aspartate receptors in rat hippocampal neurons**. *J Pharmacol Exp Ther* 2005, **312**(3):1195-1205.

199. Oliver D, Ludwig J, Reisinger E, Zoellner W, Ruppersberg JP, Fakler B: **Memantine inhibits efferent cholinergic transmission in the cochlea by blocking nicotinic acetylcholine receptors of outer hair cells**. *Mol Pharmacol* 2001, **60**(1):183-189.

200. Maskell PD, Speder P, Newberry NR, Bermudez I: **Inhibition of human alpha 7 nicotinic acetylcholine receptors by open channel blockers of N-methyl-D-aspartate receptors**. *Br J Pharmacol* 2003, **140**(7):1313-1319.

201. Pakkanen JS, Nousiainen H, Yli-Kauhaluoma J, Kylanlahti I, Moykkynen T, Korpi ER, Peng JH, Lukas RJ, Ahtee L, Tuominen RK: **Methadone increases intracellular calcium in SH-SY5Y and SH-EP1-halpha7 cells by activating neuronal nicotinic acetylcholine receptors**. *J Neurochem* 2005, **94**(5):1329-1341.

202. Fukushima H, Nagayama S, Otsuka M, Takahama K, Isohama Y, Kai H, Miyata T: **Inhibition of glycine-induced current by morphine in nucleus tractus solitarii neurones of guinea pigs**. *Methods Find Exp Clin Pharmacol* 1998, **20**(2):125-132.

203. Lioudyno MI, Verbitsky M, Holt JC, Elgoyhen AB, Guth PS: **Morphine inhibits an alpha9-acetylcholine nicotinic receptor-mediated response by a mechanism which does not involve opioid receptors**. *Hear Res* 2000, **149**(1-2):167-177.

204. Almeida LE, Pereira EF, Alkondon M, Fawcett WP, Randall WR, Albuquerque EX: **The opioid antagonist naltrexone inhibits activity and alters expression of alpha7 and alpha4beta2 nicotinic receptors in hippocampal neurons: implications for smoking cessation programs**. *Neuropharmacology* 2000, **39**(13):2740-2755.

205. Chesnoy-Marchais D, Cathala L: **Modulation of glycine responses by dihydropyridines and verapamil in rat spinal neurons**. *Eur J Neurosci* 2001, **13**(12):2195-2204.

206. Das P, Bell-Horner CL, Huang RQ, Raut A, Gonzales EB, Chen ZL, Covey DF, Dillon GH: **Inhibition of type A GABA receptors by L-type calcium channel blockers**. *Neuroscience* 2004, **124**(1):195-206.

207. Lopez MG, Fonteriz RI, Gandia L, de la Fuente M, Villarroya M, Garcia-Sancho J, Garcia AG: **The nicotinic acetylcholine receptor of the bovine chromaffin cell, a new target for dihydropyridines**. *Eur J Pharmacol* 1993, **247**(2):199-207.

208. Coyne L, Su J, Patten D, Halliwell RF: **Characterization of the interaction between fenamates and hippocampal neuron GABA(A) receptors**. *Neurochem Int* 2007, **51**(6-7):440-446.

209. Sinkkonen ST, Mansikkamaki S, Moykkynen T, Luddens H, Uusi-Oukari M, Korpi ER: **Receptor subtype-dependent positive and negative modulation of GABA(A) receptor function by niflumic acid, a nonsteroidal anti-inflammatory drug**. *Mol Pharmacol* 2003, **64**(3):753-763.

210. Ye JH, Schaefer R, Wu WH, Liu PL, Zbuzek VK, McArdle JJ: **Inhibitory effect of ondansetron on glycine response of dissociated rat hippocampal neurons**. *J Pharmacol Exp Ther* 1999, **290**(1):104-111.

211. Papke RL, Porter Papke JK, Rose GM: **Activity of alpha7-selective agonists at nicotinic and serotonin 5HT3 receptors expressed in Xenopus oocytes**. *Bioorg Med Chem Lett* 2004, **14**(8):1849-1853.

212. Sugimoto M, Fukami S, Kayakiri H, Yamazaki S, Matsuoka N, Uchida I, Mashimo T: **The beta-lactam antibiotics, penicillin-G and cefoselis have different mechanisms and sites of action at GABA(A) receptors**. *Br J Pharmacol* 2002, **135**(2):427-432.

213. Tokutomi N, Agopyan N, Akaike N: **Penicillin-induced potentiation of glycine receptor-operated chloride current in rat ventro-medial hypothalamic neurones**. *Br J Pharmacol* 1992, **106**(1):73-78.

214. Zwart R, van Kleef RG, Gotti C, Smulders CJ, Vijverberg HP: **Competitive potentiation of acetylcholine effects on neuronal nicotinic receptors by acetylcholinesterase-inhibiting drugs**. *J Neurochem* 2000, **75**(6):2492-2500.

215. Kawakami J, Yamamoto K, Asanuma A, Yanagisawa K, Sawada Y, Iga T: **Inhibitory effect of new quinolones on GABA(A) receptor-mediated response and its potentiation with felbinac in Xenopus oocytes injected with mouse-brain mRNA: correlation with convulsive potency in vivo**. *Toxicol Appl Pharmacol* 1997, **145**(2):246-254.

216. He Y, Benz A, Fu T, Wang M, Covey DF, Zorumski CF, Mennerick S: **Neuroprotective agent riluzole potentiates postsynaptic GABA(A) receptor function**. *Neuropharmacology* 2002, **42**(2):199-209.

217. Mohammadi B, Krampfl K, Moschref H, Dengler R, Bufler J: **Interaction of the neuroprotective drug riluzole with GABA(A) and glycine receptor channels**. *Eur J Pharmacol* 2001, **415**(2-3):135-140.

218. Mohammadi B, Lang N, Dengler R, Bufler J: **Interaction of high concentrations of riluzole with recombinant skeletal muscle sodium channels and adult-type nicotinic receptor channels**. *Muscle Nerve* 2002, **26**(4):539-545.

219. Chesnoy-Marchais D: **Potentiation of glycine responses by dideoxyforskolin and tamoxifen in rat spinal neurons**. *Eur J Neurosci* 2003, **17**(4):681-691.

220. Allen MC, Newland C, Valverde MA, Hardy SP: **Inhibition of ligand-gated cation-selective channels by tamoxifen**. *Eur J Pharmacol* 1998, **354**(2-3):261-269.

221. Sugimoto T, Sugimoto M, Uchida I, Mashimo T, Okada S: **Inhibitory effect of theophylline on recombinant GABA(A) receptor**. *Neuroreport* 2001, **12**(3):489-493.

222. Hara K, Minami K, Sata T: **The effects of tramadol and its metabolite on glycine, gamma-aminobutyric acidA, and N-methyl-D-aspartate receptors expressed in Xenopus oocytes**. *Anesth Analg* 2005, **100**(5):1400-1405, table of contents.

223. Mihalak KB, Carroll FI, Luetje CW: **Varenicline is a partial agonist at alpha4beta2 and a full agonist at alpha7 neuronal nicotinic receptors**. *Mol Pharmacol* 2006, **70**(3):801-805.
